# Gene expression and functional comparison between multipotential stromal cells from lateral and medial condyles of knee osteoarthritis patients

**DOI:** 10.1038/s41598-019-45820-w

**Published:** 2019-06-27

**Authors:** Clara Sanjurjo-Rodriguez, Thomas G. Baboolal, Agata N. Burska, Frederique Ponchel, Jehan J. El-Jawhari, Hemant Pandit, Dennis McGonagle, Elena Jones

**Affiliations:** 1 Leeds Institute of Rheumatic and Musculoskeletal Medicine, University of Leeds, Leeds, United Kingdom; 2 University of A Coruña, Cell Therapy and Regenerative Medicine group, Biomedical Sciences, Medicine and Physiotherapy department; CIBER-BBN, Institute of Biomedical Research of A Coruña (INIBIC)-Centre of Advanced Scientific Researches (CICA), A Coruña, Spain; 3 NIHR Leeds Musculoskeletal Biomedical Research Unit, Leeds, United Kingdom; 4 Clinical Pathology department, Mansoura University, Mansoura, Egypt; 5 Leeds Teaching Hospitals NHS Trust, Leeds, United Kingdom

**Keywords:** Osteoarthritis, Stem-cell research

## Abstract

Osteoarthritis (OA) is the most common degenerative joint disorder. Multipotential stromal cells (MSCs) have a crucial role in joint repair, but how OA severity affects their characteristics remains unknown. Knee OA provides a good model to study this, as osteochondral damage is commonly more severe in the medial weight-bearing compartment compared to lateral side of the joint. This study utilised *in vitro* functional assays, cell sorting, gene expression and immunohistochemistry to compare MSCs from medial and lateral OA femoral condyles. Despite greater cartilage loss and bone sclerosis in medial condyles, there was no significant differences in MSC numbers, growth rates or surface phenotype. Culture-expanded and freshly-purified medial-condyle MSCs expressed higher levels of several ossification-related genes. Using CD271-staining to identify MSCs, their presence and co-localisation with TRAP-positive chondroclasts was noted in the vascular channels breaching the osteochondral junction in lateral condyles. In medial condyles, MSCs were additionally found in small cavities within the sclerotic plate. These data indicate subchondral MSCs may be involved in OA progression by participating in cartilage destruction, calcification and sclerotic plate formation and that they remain abundant in severe disease. Biological or biomechanical modulation of these MSCs may be a new strategy towards cartilage and bone restoration in knee OA.

## Introduction

Osteoarthritis (OA) is the major cause of chronic pain and disability and its incidence is predicted to increase in coming decades^1,2^. Historically described as a degenerative disease of cartilage, OA is now viewed as a disease of the whole joint with characteristic abnormalities to subchondral bone, and no disease-modifying drugs available to induce remission^3^. Abnormalities in OA bone such as subchondral plate sclerosis and bone marrow lesions (BMLs) are anatomically ‘mapped’ to the areas of abnormal joint loading and cartilage denudation^4^, suggesting that the disease process affects the osteochondral unit as a whole, rather than cartilage or bone separately^5–8^.

In a healthy osteochondral unit, some cross-talk between cartilage and subchondral bone cells occurs for balanced mechanotransduction and nourishment of deep cartilage layers. Multipotential stromal cells (MSCs), the precursors of both osteocytes and chondrocytes, are present in the subchondral bone^9^ but also in the surrounding synovium and synovial fluid (SF)^10^, with early cartilage lesions likely to be repaired by joint resident MSCs^11^. However, in OA, damage to the osteochondral junction may lead to altered molecular exchanges and activation of subchondral bone MSCs^5,12^.

We have previously shown the presence of MSCs in healthy and arthritic SF, and their numerical increase in OA compared to arthroscopically normal joints^10^. However, despite their apparent increase as the disease progresses^13^, cartilage lesions in OA continue to deteriorate suggesting that locally available SF MSCs have reduced joint repair potentials, possibly due to their impaired attachment to cartilage^14^, reduced chondrogenic ability^15^ or persistent abnormal joint mechanical loading^4^. MSCs resident in OA subchondral bone may also be affected by the osteoarthritic process, this time as a result of altered joint biomechanics or the breached osteochondral junction. For example, using a mouse anterior cruciate ligament transection (ACLT) model of OA, Zhen *et al*. have recently documented an abnormal recruitment and osteogenic commitment of subchondral bone MSCs leading to aberrant bone formation and BMLs^16^. In agreement, we previously demonstrated that MSCs were more numerous in subchondral BMLs in late-stage hip OA patients^17^ and had gene expression bias towards bone formation as opposed to bone resorption^17^, compared to MSCs from the non-BML areas. As MSCs are the earliest precursors of osteoblasts and chondrocytes, these abnormalities in subchondral bone MSCs could be directly responsible for the formation of sclerotic bone later in disease^18^, instead of rebuilding new cartilage. Present immediately beneath chondral lesions^17^, these osteogenically-committed MSCs could also influence the remaining cartilage metabolism. Whether the same MSC behaviour can be seen in knee OA subchondral bone MSCs, remains unknown.

Human knee OA provides a potentially unique model for the study of MSCs in joint repair, homeostasis and OA progression. This is because the anatomical structure and biomechanical configuration of the knee is typically associated with much more severe cartilage loss on the predominantly weight-bearing medial compartment^4,19–21^. The aim of this study was therefore to investigate and compare the numbers, topography and gene expression profiles of subchondral bone-resident MSCs from medial and lateral femoral condyles from the same patient. We additionally hypothesised that due to increased molecular exchanges through damaged osteochondral junction, parallel gene expression alterations can be seen in medial condyle chondrocytes. As gene expression in bone-resident MSCs is known to be affected by culture expansion^22–24^, the analysis was performed on MSCs directly taken from their native environment as well as following culture expansion.

## Results

### Histological assessment of tissue damage in medial and lateral femoral condyles

**Figure 1:**
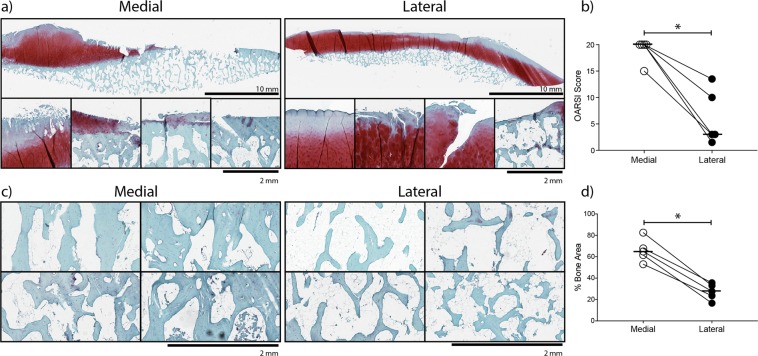
Histological assessment of tissue damage in osteoarthritic femoral condyles. (**a**) Example macroscopic (top) and microscopic (bottom) images of safranin-O stained sections illustrating the spectrum on cartilage damage in both medial and lateral condyles. In the medial condyles microscopic images show regions from different donors ranging from surface fibrillation and fissures with cartilage matrix loss (far left) to complete cartilage loss and sclerotic plate formation (far right). In lateral condyles, microscopic images show matrix loss with some surface abrasion (far left) to complete cartilage loss (in a valgus patient) with no evidence for bone sclerosis). (**b**) OARSI grading of the whole medial and lateral condyle surfaces showing significantly less cartilage damage in lateral condyles. (**c**) Example microscopic images of subchondral bone from different donors illustrating the increased bone anabolic response to loss of cartilage on the medial compared to the lateral condyle. (**d**) Bone area analysis of these areas demonstrating the increase in bone area as a percentage of total area in the medial compartment. *p < 0.05, n = 5 donors. Horizontal bars show medians.

Whole medial and lateral condyle specimens were decalcified in EDTA and the cartilage damage assessed using the OARSI OA cartilage histopathology scoring system^25^ (Fig. 1a,b). Four out of five medial condyles had OARSI score of 20 indicating maximum damage characterized by denuded bone in more than 50% of the specimen surface. Lateral condyles were more variable, with cartilage damage scores ranging from 1.5 to 15 (15 corresponding to the one valgus deformity patient in our cohort) (Fig. 1b). Superficial fibrillation or simple fissures in the outer cartilage areas were most commonly observed in lateral condyles, however, a focal area of denuded bone was seen in the valgus donor (Fig. 1a, lateral). As expected, subchondral bone area measured as a percentage of total area was higher in medial compared with lateral condyles (Fig. 1c,d), and the highest in the sclerotic areas that were fully denuded of cartilage (Fig. 1c, medial). Altogether these data were consistent with previous findings indicating much more pronounced osteochondral damage and therefore more severe disease in medial condyles of patients with a varus deformity of the knee^19,21^.

### Comparison of subchondral MSC numbers and *in vitro* functional capacity between medial and lateral condyles

**Figure 2:**
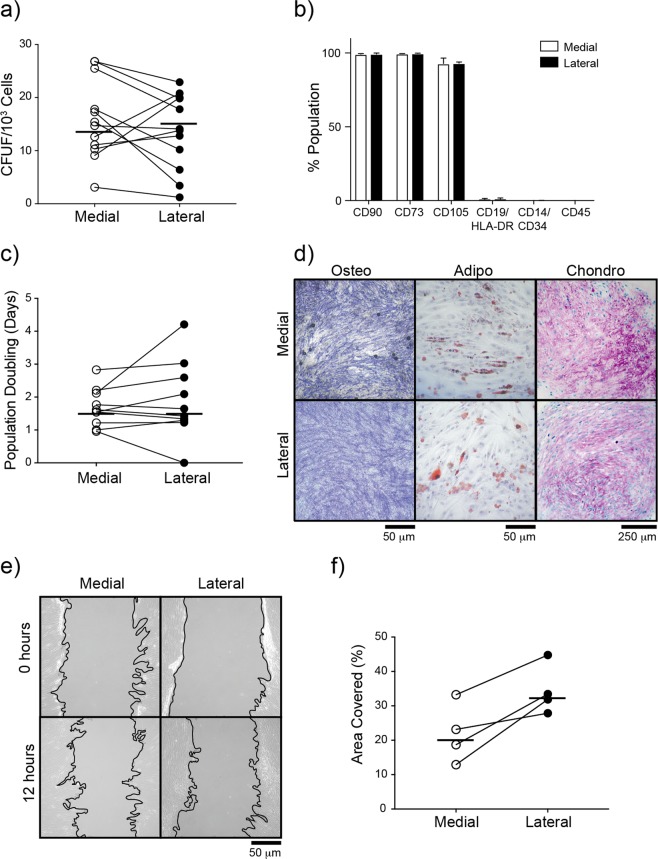
Functional analysis of subchondral bone MSCs from medial and lateral condyles. (**a**) Comparison of MSC frequency measured as a proportion of colony forming units fibroblast in relation to total enzymatically-extracted cells for donor matched samples. (**b**) Phenotypic profile of culture expanded medial and lateral condyle derived MSCs indicating no differences in the expression of standard MSC markers. (**c**) Comparison of growth rates of medial and lateral condyle derived MSCs measured as population doubling times (in days) for donor matched samples. (**d**) Example images of differentiation assays performed with donor matched samples. Osteogenic images (Osteo) show positive alkaline phosphatase staining on day 14 post osteogenic induction, adipogenesis assay (Adipo) shows accumulation of Oil Red-O stained lipid vesicles on day 14 post adipogenic induction and toluidine blue staining of chondrogenic pellet cultures (Chondro) shows accumulation of proteoglycans (purple) on day 21 post chondrogenic induction. (**e**) Examples migration assay images from baseline (top) and 12 hours post scratch (bottom) wound for medial and lateral MSCs. Black line indicates the migrating front of the cells. (**f**) Analysis of migration assay images for donor matched medial and lateral MSCs showing the percentage of wound coverage by migrating cells after 12 hours (relative to the corresponding 0 hour area), and showing a trend for higher motility by lateral condyle MSCs.

In a cohort of 11 of varus patients, analysis of the colony-forming capacity of cell extracts obtained from collagenase digestion, revealed no difference in the frequency of MSCs measured as a percentage of CFU-Fs in relation to total extracted cells (Fig. 2a). Similarly, no difference was found in the numbers of CFU-F per gram of bone (data not shown). Cultures generated from the extracted cells possessed standard MSC surface phenotype, with no differences in CD73, CD90 and CD105 expression levels (Fig. 2b), and exhibited similar growth rates (Fig. 2c). No trends for a differentiation bias between medial and lateral condyle MSCs were found following tri-lineage differentiation, however large patient variability was observed. Differentiation results for one patient are shown in Fig. 2d.

Interestingly, using a scratch migration assay, lateral condyle MSCs from all patients showed slightly higher motility rates (measured as percentage of scratched area covered by cells after 12 hours, (Fig. 2e,f) whereas their angiogenesis-support capability appeared to be similar (Supplementary Fig. 1).

### Gene expression differences between medial- and lateral condyle MSCs

**Figure 3:**
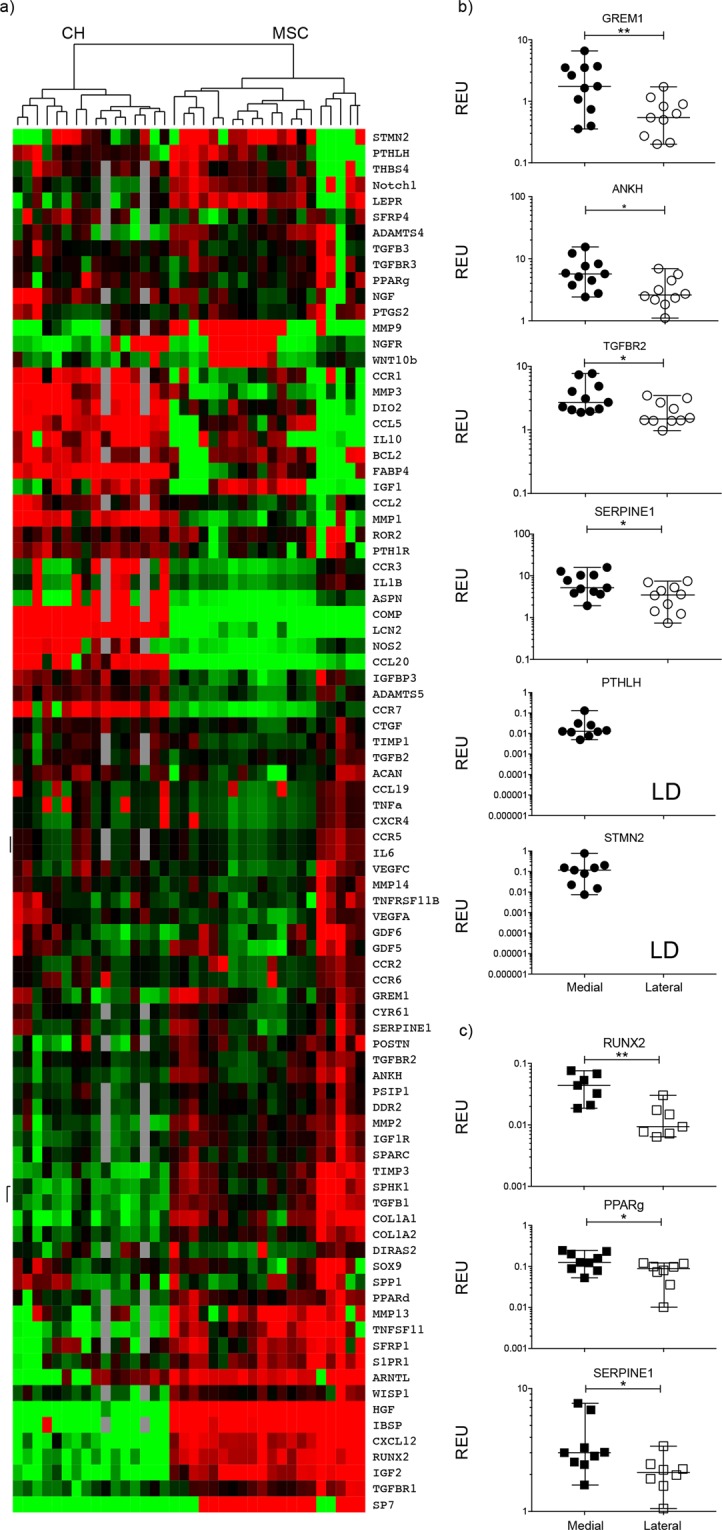
Gene expression analysis of culture expanded, medial and lateral condyle derived MSCs and chondrocytes. (**a**) Cluster analysis between chondrocytes (CH) and MSCs from both the medial and lateral femoral condyles illustrating clear clustering of MSCs away from chondrocytes. Data were normalized to the housekeeping gene HPRT and log2 transformation and data filtering were performed according to standard methods described in Churchman *et al*.^22^. Scores were assigned as follows: black = 1, red > 1, green < 1; grey = missing data (below detection). (**b**) Differentially expressed genes between medial and lateral MSCs with statistically significant differences in donor-matched and non-matched analysis. LD indicates transcripts which were rarely expressed (in < 50% samples). (**c**) Differentially expressed between medial and lateral chondrocytes with statistically significant differences in donor-matched and non-matched analysis. *p < 0.05; **p < 0.01. Horizontal bars show medians. REU: relative expression units (relative to housekeeping HPRT).

Gene expression (for 95 transcripts selected based on their involvement in MSC tri-lineage differentiation, chondroprotection, cartilage metabolism, genetic association with OA, or OA progression^17,26–30^) was first compared between culture expanded MSCs and chondrocytes to assess whether the selected gene panel reflected the cell identity/function. Irrespective of their condyle origin, cultured MSCs and chondrocytes separated from each other when gene expression profiles were analyzed using an unsupervised hierarchical clustering approach (Fig. 3a), with 30 and 18 genes expressed at higher level in MSCs and chondrocytes, respectively (Supplementary Table 1). Of note, highly expressed genes in MSCs included classical bone structural molecules such as *IBSP* (bone sialoprotein) and *COL*1*A1* (collagen type I alpha 1 chain), while structural cartilage protein *COMP* (cartilage oligomeric matrix protein) was only expressed in chondrocytes (Supplementary Table 1).

The analysis was next performed between medial and lateral condyles MSCs from the varus deformity patients (Fig. 3b). Not all of the genes were expressed in all samples, therefore an unpaired statistical analysis was first performed, followed by paired tests when possible (i.e. when > 6 pairs could be used). Four genes *GREM1* (gremlin1), *ANKH* (inorganic pyrophosphate transport regulator), *TGFBR2* (transforming growth factor beta receptor 2) and *SERPINE1* (serpin family E member 1) out of 95 were found to be higher in medial condyle MSCs (p < 0.05 for all 4 genes, using both unpaired and paired tests, Fig. 3b). The largest differences were found for *GREM1* (median fold 2.9) and *ANKH* (median fold 2.1). Additionally, *STMN2* (stathmin 2) and *PTHLH* (parathyroid hormone like hormone) displayed low detection frequency in lateral condyle MSCs indicating strong differential expression (Fig. 3b). Interestingly, low *PTHLH* detection frequency in lateral condyles MSCs was preferentially seen in female patients compared to male patients (detected in 2 out of 6 females compared to 4 out of 5 males). None of the genes tested showed significantly higher expression in lateral condyle MSCs.

### Gene expression differences between medial- and lateral condyle chondrocytes

To investigate if differentially-expressed genes were specific to MSCs or also observed in chondrocytes thus indicating co-regulation in both cell types, gene expression levels were compared between medial and lateral condyle chondrocyte cultures of the varus deformity patients (Fig. 3c). A different set of genes was differentially expressed, with 3 genes over-expressed in medial condyle chondrocytes: *RUNX2* (runt-related transcription factor 2), *PPARg* (peroxisome proliferator activated receptor gamma) and *SERPINE1* (Fig. 3c) while again, none were found to be higher in lateral condyle chondrocytes. Altogether, only one gene, *SERPINE1*, showed a similar differential expression between the condyles in both MSCs and chondrocytes.

### Gene expression differences between uncultured medial- and lateral condyle CD45^−^CD271^+^ MSCs

**Figure 4:**
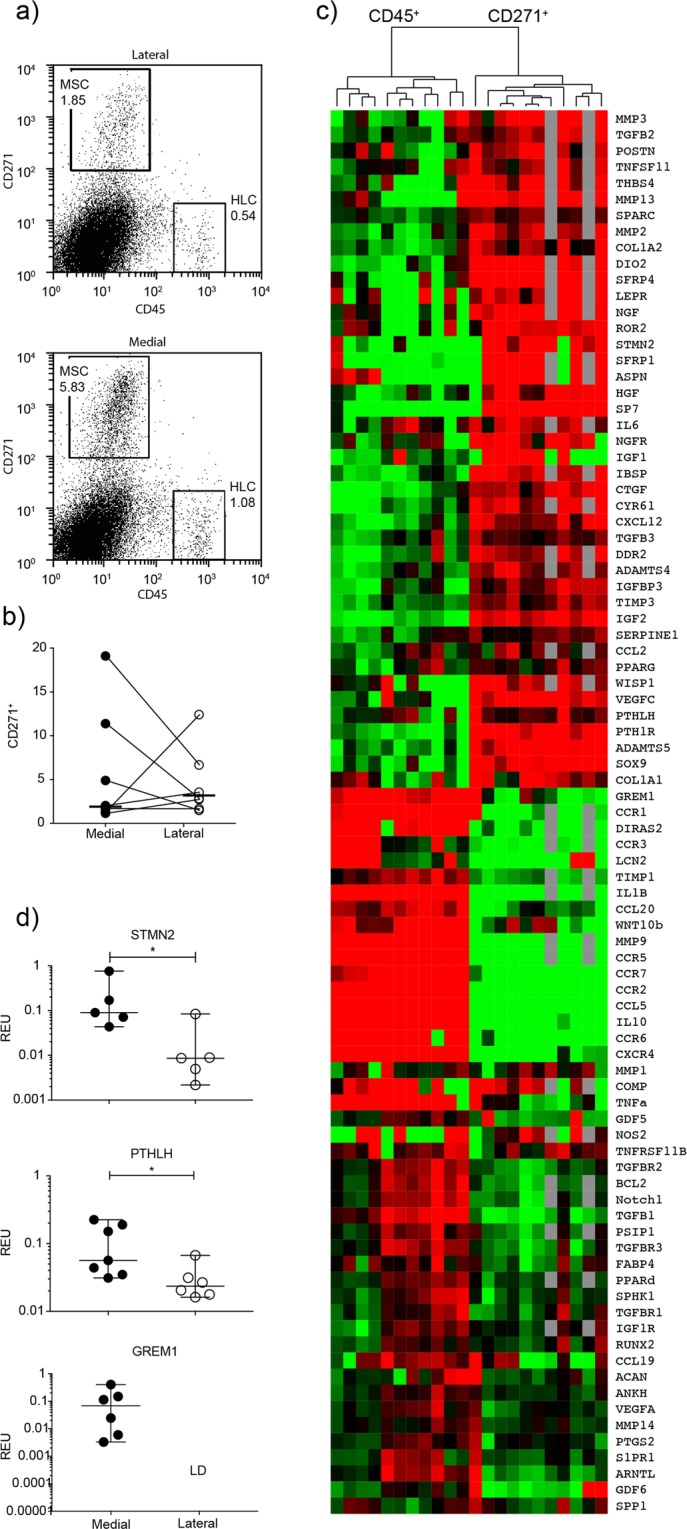
Native subchondral bone MSCs gene expression analysis. (**a**) Flow cytometry dot plots illustrating sorting gates for CD45^-^CD271^+^ MSCs and CD45^+^ CD271^-^ haematopoietic lineage cells (HLCs) together with representative frequencies of MSCs and HLCs measured as a percentage of total live cells (square boxes) for donor matched medial and lateral condyles. (**b**) Comparison of CD45^-^ CD271^+^ MSC frequency for donor matched samples measured by flow cytometry. Y-axis represents CD45^-^ CD271^+^ cells as percentage of total live cells. (**c**) Cluster analysis between CD45^ +^ HLCs and CD271 ^+^ MSCs from both the medial and lateral femoral condyles, and illustrating clear clustering of MSCs away from HLCs. Data were normalized to the housekeeping gene HPRT and log2 transformation and data filtering were performed according to standard methods described in Churchman *et al*.^22^. Scores were assigned as follows: black = 1, red > 1, green < 1; grey = missing data (below detection). (**d**) Selected genes with differential expression patterns between medial and lateral condyle derived CD45-CD271^ +^ native MSCs. LD indicates transcripts which were rarely expressed (in < 50% samples). *p < 0.005. Horizontal bars show medians. REU: relative expression units (relative to housekeeping HPRT).

It has been documented that even minimal expansion of MSCs in standard conditions leads to changes in gene expression^22,31,32^. To investigate if gene expression differences between medial and lateral condyle MSCs identified using cultured cells were also measurable *in vivo*, we proceeded with gene expression analysis on CD45^−^CD271^+^ sorted cells (Fig. 4a), a recognized phenotype of native human bone-resident MSCs^33,34^ from the varus deformity patients. Consistent with CFU-F data (Fig. 2a), no statistically significant differences in the proportions of CD45^−^CD271^+^ cells was found between medial and lateral condyles’ tissue digests (Fig. 4b).

CD45^+^CD271^−^ hematopoietic lineage cells (HLCs) sorted from the same tissue digests (Fig. 4a) were used as a negative control population and clustered separately from CD45^−^CD271^+^ MSCs (Fig. 4c). As expected, genes involved in immune response and cell migration/chemotaxis (for example, *IL1B* (interleukin 1 beta) and *CCL5* (C-C motif chemokine ligand 5)) were expressed at higher levels in CD45^+^CD271^−^ HLCs (Supplementary Table 2), while genes involved in mesenchymal lineage differentiation (for example, *SP7*, *SOX9* (SRY-box 9) and *PPARg*) were expressed at higher levels in CD45^−^CD271^+^ MSCs (Supplementary Table 2).

When the levels of expression were compared between medial and lateral condyle CD45^−^CD271^+^ MSCs, most of the genes did not show differential expression but three genes were significantly higher in medial condyle MSCs: *GREM1*, *STMN2* and *PTHLH* (Fig. 4d), all of which were first found on our cultured MSC screen (Fig. 3b). Again, no genes were found to be higher in lateral CD45^−^CD271^+^ MSCs.

### The topography of CD271^+^ MSCs in the osteochondral areas of medial and lateral condyles

**Figure 5:**
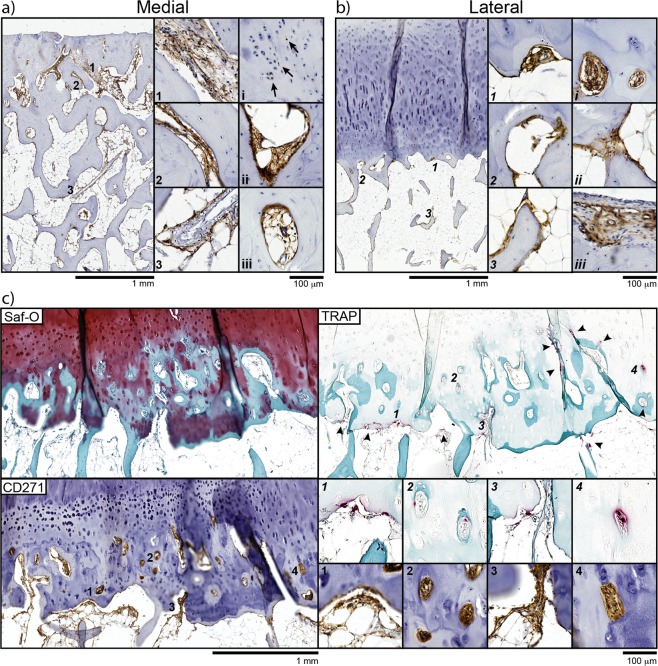
Topography of CD271^+^ MSCs in OA femoral condyles. (**a**) Immunohistochemistry of medial condyle joint surface showing the distribution of CD271^+^ MSCs within the sclerotic area of denuded bone. Magnified images show corresponding areas of CD271 staining in stromal tissue (1), on bone lining (2) and in perivascular regions (3). Additional images from different donors show isolated CD271 positivity in chondrocytes (i, arrows), bone lining (ii) and perivascular (iii) staining. (**b**) Immunohistochemistry of the lateral condyle joint surface showing the distribution of CD271^+^ MSCs in the lateral condyle. Magnified images shown corresponding (1–3) areas and features from other donors shown in (i-iii). Vascular channels invading the cartilage (*1* and *i*) were a common feature in all donors as was the typical bone lining (*2*, *3* and *ii*) appearance of CD271^+^ MSCs. No CD271 was seen in chondrocytes, however some positivity was seen in stromal tissue in areas of cartilage damage (iii). (**c**) Adjacent Safranin O (Saf-O), TRAP and CD271 stained tissue sections showing co-localisation of TRAP + chondroclasts and CD271^+^ MSCs in a region of abundant vascular/bone invasion into the articular cartilage. Safranin-O staining shows areas of GAG depletion and bone invasion (blue). The corresponding CD271 and TRAP images indicate that these cartilage erosion and bone formation processes may be mediated by chondroclasts and neighboring MSCs, respectively. Magnified images show specific TRAP and CD271 co-localisation with arrow heads highlighting additional TRAP + cells.

Although we did not find significant differences between medial and lateral condyles in the proportions of CFU-Fs (Fig. 2a) or CD45^−^CD271^+^ cells (Fig. 4b), following their enzymatic extraction from the subchondral bone, it is possible that damage-specific topographical niches for these cells could be found *in situ* (Fig. 5). In the medial condyles, where large (>50%) areas of the articulating surface displayed fully denuded bone, CD271^+^ cells were found in the remaining bone cavities of the sclerotic plate, typically in stromal, bone-lining and perivascular regions (Fig. 5a, areas 1, 2 and 3, respectively). Strong CD271 positivity was also found in the stromal tissue within occasional subchondral cysts (Supplementary Fig. 2). In the lateral condyles of the varus knees, CD271^+^ cells were commonly observed within vascular channels invading into the articular cartilage (Fig. 5b, areas 1 and *i*), as well as lining trabeculae (area 3). Interestingly, CD271^+^ cells also were often found near TRAP+ chondroclasts, and parallel Safranin O staining of adjacent sections clearly indicated cartilage replacement by new bone in these particular areas (Fig. 5c). This ‘vascular channel’ MSC topography was also seen in less damaged areas of the medial condyles, directly underneath any remaining articular cartilage and likely reflecting a common early process in the OA knee joint, where articular cartilage still remains and covers the bone (not shown). Similar immunohistological findings were also observed in the proximal tibial plateau samples from the varus knees (Supplementary Fig. 3).

## Discussion

This study evaluated numerical, topographical and gene expression changes in subchondral bone MSCs in knee OA, in relation to osteochondral damage progression, by comparing the medial and lateral femoral condyles from the same patients with varus deformity knee OA. This is the most common type of knee OA, in which the mechanical load is preferentially transmitted to the medial compartment of the knee^35,36^. In a rarer valgus deformity type, the load-bearing axis passes lateral to the knee, and the resulting force increases on the lateral compartment^36^. Our data reveal no significant differences in subchondral bone MSC numbers between medial and lateral condyles of varus patients, as well as their comparable growth rates and trilineage capacities *in vitro*. A broadly similar pattern of MSC topography was seen in both condyles with their notable presence in the vascular channels breaching the osteochondral junction in early lesioned areas and within subchondral bone cavities. However, medial condyles MSCs were also present in small sclerotic plate cavities, and had notable gene expression alterations, first identified in *in vitro*-expanded MSC, and then confirmed using uncultured sort-purified CD45^−^CD271^+^ MSCs. To our best knowledge, this is therefore the first study to describe the topographies and identify novel genes specifically up-regulated in MSCs from more-damaged, sclerotic subchondral bone areas in human knee OA.

Gene expression data identified *STMN2*, *PTHLH* and *GREM1* as the three most consistent upregulated genes in the more damaged, medial condyle MSCs screened on both *in vivo* and in culture-expanded MSCs. These genes were not differentially expressed in chondrocytes or HLCs indicating that the observed differences were MSC-specific. *GREM1* has been recently shown as a marker of osteochondral skeletal stem cells in mice^37^, and involved in the regulation of chondrocyte hypertrophy, osteogenic differentiation and endochondral ossification^38,39^. *PTHLH* has been initially identified as OA susceptibility gene, and later shown to be involved in the regulation of both bone and cartilage development^40,41^, including chondrocyte hypertrophy^42–45^. *PTHLH* gene encodes parathyroid hormone related protein (PTHrP) which has multiple functions including its anabolic activity on bone cells, and is used for osteoporosis treatment^46^. Its reduced expression in more OA damaged condyles, and particularly in female patients, suggests that *PTHLH* can be a good candidate gene for further analysis in larger, gender split patient cohorts.

Most exciting, is our finding of an up-regulation in medial condyle MSCs of *STMN2*, a small phosphoprotein involved in the neuronal microtubule dynamics, proliferation, differentiation and migration^47,48^, and upregulated by nerve growth factor^49^. Its role in skeletal tissue development was first demonstrated by Chiellini *et al*.^49^ who showed that STMN2 was expressed by MSCs that undergone osteogenic but not adipogenic differentiation. A recent study has also documented the significant up-regulation of *STMN2* in the BML regions of knee OA patients^26^, complementing our present findings. Another gene, *ANKH* was upregulated in medial condyle cultured MSCs, and narrowly missed significance in uncultured CD45^−^CD271^+^ cells. This molecule also plays an important role in the regulation of differentiation and mineralization in both bone and cartilage^50^.

Altogether, this gene expression pattern indicates potential MSC involvement in the processes of cartilage calcification, bone formation and mineralisation, supporting previous observations^16,51^ and our histological findings on the topography of CD271^+^ cells *in situ* where such cells remained present in sclerotic plate cavities. As a broadly similar pattern of MSC topography was seen in medial tibial plateaus and in our hip OA study^17^, this indicates subchondral bone MSC involvement in the disease progression in both major types of human OA. While no up-regulated genes were found in lateral condyle MSCs based on the gene panel selected for this study, our histological observations showed the presence of CD271^+^ MSCs in the vascular channels that migrate upwards and breach the osteochondral junction in early disease stages, which were more apparent in lateral condyles. This is in agreement with our functional data indicating higher motility of lateral compared to medial condyle MSCs.

Based on animal model studies^16^, and our previous hip OA findings^17^, we expected to detect higher numbers of MSCs in more damaged, medial condyles. Although histological assessment of cartilage damage confirmed an overall more damaged status of medial condyles in varus patients^4,20,52^, both medial and lateral condyles showed regional variations in the amount of damage, which may have affected our results. A more selective, ‘region-based’ approach, as undertaken in recent microarray studies^28,29,53^, may have been more accurate, but this was deemed technically unattainable due to the small amounts of bone available for subsequent isolation and sorting of rare MSCs. However, in contrast to the above-mentioned microarray gene expression studies^26,28,29,53^, we were able to assign up- or down-regulated gene expression to specific cell types, i.e. MSCs or HLCs further clarifying the role of MSCs in OA pathogenesis.

Finally, we used cultured MSCs and chondrocytes to test a hypothesis of gene expression co-regulation in OA^28^, as a result of increased molecular exchange through the breached osteochondral junction. Whereas a little overlap between differentially regulated genes in both MSCs and chondrocytes was found, *SERPINE1* was one molecule showing parallel up-regulation in medial condyle MSCs and chondrocytes. Consistent with our findings SERPINE1 protein was found to be more abundant in the supernatants of cultured osteoblasts derived from sclerotic areas of OA subchondral bone^54^, however in contrast to our study, its mRNA expression was found to be lower in medial condyle chondrocytes, compared to healthy or lateral condyle chondrocytes in another study^55^. These differences can be partially explained by the fact, that *SERPINE1* expression is downregulated in chondrocyte culture^56^.

In summary, this study documents the presence, topography and gene expression profile of the subchondral bone MSCs in femoral condyles of the most common, varus type of human knee OA. It showed that even in late disease stages, there is no obvious numerical MSC defect on the most worn medial joint compartment, however medial condyle MSCs had characteristic upregulation of several ossification-related genes. Further studies are needed to establish whether these differences in MSCs are due to an ongoing, albeit aberrant, tissue repair process or a reaction to mechanical over-stress due to alterations in prevalent mechanical forces. These questions can be further addressed by looking at the topography of MSCs in relation to a newly-formed osteoid or early osteocytes, as shown in our recent study in hip OA patients^57^, and using larger cohorts of varus and valgus OA patients. As shown here, native subchondral bone MSCs remain present in late disease stages and are therefore available for biomechanical or therapeutic targeting, and we identified several gene candidates such as *GREM1*, *PTHLH* and *STMN2*, for such targeting.

Further work is also needed to establish if biomechanical (such as knee joint distraction^14,58^) or biological stimulation of these MSCs (such as direct or extracellular vesicle-based microRNA modulation)^59,60^ can result in gene expression changes in preference of cartilage tissue formation and subchondral bone restoration. Also, a better understanding of cellular and molecular events leading to vascular channel formation and upward migration in early disease stages, and the role of MSCs in these processes, could lead to novel therapies for early OA.

## Materials and Methods

### Patient samples

Ethical approval for this study was obtained from the Yorkshire & The Humber - South Yorkshire Research Ethics Committee (14/YH/0087), in compliance with the Helsinki Declaration of ethical principles for medical research involving human subjects. Seventeen patients (median age 72 years old, range 56–83) were included in this study after signing informed consent, who all underwent total knee arthroplasty. Of these patients, all but two had a varus deformity indicating the medial aspects of the joint were the prominent weight bearing surface. All the patients met the American College of Rheumatology criteria for knee osteoarthritis (OA) (Altman, 1986) and gave written informed consent. Exclusion criteria were: history of inflammatory arthritis, metastatic cancer or other disorders affecting bone.

From all arthroplasties, intact femoral condyle samples were collected in saline and transferred to the laboratory immediately upon receipt.

### Histology and immunohistochemistry

Whole condyles from five knee replacement surgeries (four with varus and one with valgus deformity) were fixed in 3.7% formaldehyde (Thermofisher Scientific, UK) for 1 week, decalcified using 0.5 M ethylenediaminetetraacetic acid (EDTA; Sigma, USA) for at least 6 months (dental x-rays used to assess extend of decalcification), re-fixed in formaldehyde for further 2 days before embedding in paraffin blocks, as described for tibial plateaus^51^. Safranin O-Fast Green (SO-FG) and Tartrate-resistant acid phosphatase (TRAP) staining were performed on 5–7 µm thick sections of the whole condyles, according to standard protocols (all reagents from Sigma). Stained slides were scanned using Leica Aperio AT2 (Leica, Germany) and images were taken with Aperio Imagescope (Leica).

For each pair of condyles, the ratio of bone area to total area of subchondral bone was assessed on SO-FG images using ImageJ 1.51t software (National Institutes of Health, USA), as previously described^17^. Briefly, at least 4 non-overlapping areas (5 mm width × 1 mm height) directly underneath the cartilage were analysed and averaged, and the bone area was calculated as a percentage of total area (mean ± SD). Cartilage damage across the whole condyle (sagittal plane) was scored by agreement of two independent observers using the OARSI OA cartilage histopathology scoring system^25^. This scoring system is widely used^16,61,62^ for microscopic assessment as it takes into account both the severity and the extent of the lesion over the measured surface, which in this study was over the whole femoral condyle. TRAP-positive chondroclasts were also identified by two independent observers, as previously described^63^.

Immunohistochemical staining for CD271-positive cells, the native MSC phenotype in bone^33,34,64,65^, was performed using monoclonal mouse anti-human antibody (clone ME20.4; Invitrogen, USA) at 1:200 dilution followed by incubation with horse radish peroxidase (HRP) and 3,3′-diaminobenzidine tetra hydrochloride (DAB) from the EnVision + Dual Link System-HRP (DAB+) (Dako, Agilent, USA), as previously described^17^. Sections were counterstained with hematoxylin and the slides scanned using Leica Aperio AT2.

### Isolation and culture of MSCs and chondrocytes

Using varus patients samples, articular cartilage was harvested from lateral and medial condyle surfaces using a scalpel, and chondrocytes were isolated as described before^66^. Briefly, cartilage was minced using a scalpel and digested overnight with 3,000 units of collagenase/g of tissue (Worthington Biochem Corp, USA). Homogenate was filtered with 22 µm cell strainer (Corning Inc., USA) before centrifugation at 450 × g for 10 minutes, followed by 5 minutes of 1x trypsin digestion. Chondrocytes were expanded in Dulbecco’s Modified Eagle’s Medium (DMEM; ThermoFisher Scientific, USA) with 10% fetal bovine serum (FBS; BioSera, France) and 1% penicillin/streptomycin (P/S; ThermoFisher Scientific). Media were changed twice a week and subculture was performed when cells reached 80% confluence, and passaged to p1.

For MSC isolation, the remaining subchondral and trabecular bone after removal of cartilage was weighted and minced mechanically into small fragments with a rongeur and digested for four hours with 3,000 units of collagenase/g, as previously described^17^. The liquid fraction was then filtered through 22 µm cell strainer before centrifugation at 450 × g for 10 minutes to pellet the extracted cells. Following counting, the cells were used for CFU-F assay, culture expansion or frozen in liquid nitrogen for subsequent cell sorting experiments. MSC cultures were established by seeding 10^6^ cells in 25 cm^2^ flasks (Corning Inc.) containing StemMACS™ MSC Expansion Media (Miltenyi Biotec, Germany) supplemented with 1% P/S. Media were changed twice a week, cells were split when reached 80% confluence and passaged up to p5.

### CFU-F assay

To enumerate MSCs, the colony-forming unit-fibroblast (CFU-F) assay was performed using freshly-digested cells seeded at a density of 5 × 10^3^ into 60 mm culture dishes (Corning Inc.) with StemMACS™ MSC Expansion Media in duplicate. Media was changed twice a week and after 10 days, the dishes were fixed with 3.7% formaldehyde and stained with methylene blue to visualize colonies, as previously described^67^. Colony numbers from duplicate dishes were counted, averaged and converted to CFU-F/10^6^ cells (or per gram of bone), and compared between medial and lateral condyles for each patient.

### Purification of uncultured subchondral bone MSCs by cell sorting

Cell sorting was performed using frozen/thawed cells obtained directly after bone digestion, as described before^9,22^. In brief, cells were thawed, re-suspended in fluorescent activated cell sorting (FACs) buffer and incubated in a blocking buffer (Miltenyi Biotec, UK) for 10 minutes before staining with the following antibodies: CD271 (allophycocyanin, APC; Miltenyi Biotec) and CD45 (Fluorescein-5-isothiocyanate, FITC; BD Biosciences, UK). Dead cells were excluded following staining with 7-AAD (Sigma) immediately prior to sorting. Cell populations: MSCs (CD45^−^CD271^+^) and control haematopoietic lineage cells (HLCs) (CD45^+^CD271^−^) were sorted into 2 separate tubes containing lysis buffer for RNA isolation (Norgen Bioteck Corp., Canada), using a BD Influx cell sorter (BD Biosciences, USA).

### Gene expression analysis

RNA was isolated from sorted CD45^−^CD271^+^ MSCs and control HLCs (donor-matched samples from lateral and medial condyles), using the total RNA Purification kit (Norgen Bioteck Corp.), following manufacturer’s instructions. Also, RNA was isolated from donor- and condyle matched passage 1 (p1) MSCs (thereafter referred to as cultured MSCs/cMSCs) and p1 chondrocytes (CH). Gene expression was performed using the 48.48 IFC (Integrated Fluidic Circuit), using recommended reagents (Fluidigm Corporation, USA), and standard TaqMan Assays (Thermofisher Scientific) following manufacturer’s recommendations. The 95 genes of interest were chosen based on their involvement in MSC trilineage differentiation, chondroprotection, cartilage metabolism, genetic association with OA, or OA progression as identified from genome-wide screens^17,26–28,30,53^ (Supplementary Table 3). Hypoxanthine Phosphoribosyltransferase 1 (HPRT1) was used as a housekeeping gene to calculate relative expression values.

Reverse transcription was performed to convert RNA to cDNA followed by 12 pre-amplification cycles for sorted and cultured cells, using a mixture of 96 Taqman gene expression assays (Supplementary Table 1). Diluted pre-amplified cDNAs were mixed with sample loading buffer (Fludidigm Corporation) and Taqman Universal PCR Master mix (Applied Biosystems, USA) before transferring to the Dynamic 48.48 IFC sample compartment. Each of the 96 Taqman assays were independently mixed with assay loading buffer (Fludidigm Corporation) and transferred to the wells of the relevant compartment on the IFC. The IFC was then run on the Biomark Real Time PCR System using a GE 48 × 48 Standard v1 PCR thermal protocol. Data was analysed using BioMark Gene Expression Data software and normalized to the housekeeping gene. Genes differentially expressed between MSCs, HLCs and chondrocytes were further scrutinized for hierarchical clustering analysis using Cluster 3.0 software and Java TreeView^22^. For cluster analysis, only genes expressed in ≥80% of samples and cultures who expressed ≥60% of all genes were included. Individual protein information and protein network analysis was obtained using the String database^68^.

### Tri-lineage differentiation assays

Osteogenic, chondrogenic and adipogenic capacities of medial and lateral condyle MSCs were assessed and compared, as previously described^10^. In brief, MSCs were each seeded in triplicate wells of 24 well-plates (Corning) for osteogenic and adipogenic differentiation. Osteogenic media contained DMEM supplemented with 10% FBS, 100 nM dexamethasone, 0.05 mM ascorbic acid and 100 mM β-glycerosphosphate (all from Sigma). Cells were cultured and osteogenic differentiation was assessed by Alkaline phosphatase activity staining.

MSC adipogenic differentiation was assessed by Oil Red O staining, after culture in adipogenic media: DMEM supplemented with 12.5% FBS, 12.5% horse serum, 0.5 mM isobutylmethylxanthine, 60 µM indomethacin and 0.5 mM hydrocortisone (all from Sigma). Chondrogenic differentiation was performed in 3D-pellet culture in ChondroDiff media (Miltenyi Biotec) and glycosaminoglycan (GAG) content was evaluated in 5μm sectioned pellets stained with Toluidine Blue^17^.

### MSC motility assay

*In vitro* motility of MSCs was assessed using a scratch assay as described before^69^. Briefly, MSCs were grown to confluence in a 6-well plate in StemMACS culture media. After removing the media, the cell monolayer was scratched with a sterile 200 µl pipette tip and washed with PBS before replacing with StemMACS™ MSC Expansion Media. At least 3 images along the open scratch were taken at 0 and 12 hours. Measurements along the scratch were averaged and percentage area closed was calculated using ImageJ software, normalized to time 0, and compared between medial and lateral condyles.

### Statistics

Results were analysed using Mann-Whitney and Wilcoxon Signed Rank tests for unpaired and paired data, respectively. The statistical analysis was performed using Prism software (version 7.0 a; GraphPad). The difference between the groups was considered as statistically significant if the p value < 0.05.

## Supplementary information

Supplementary material

## Data Availability

The gene expression array data that support the findings of this study is available from the authors upon request.
