# Supplementary material for: Gene expression and functional comparison between multipotential stromal cells from lateral and medial condyles of knee osteoarthritis patients
Source: Sci Rep. 2019 Jun 27;9:9321. doi: 10.1038/s41598-019-45820-w (PMC6597541; doi:10.1038/s41598-019-45820-w)
Supplement: Supplementary file 1 — Supplementary material [file 41598_2019_45820_MOESM1_ESM.docx]

**Supplementary material**

**Gene expression and functional comparison between multipotential stromal cells from lateral and medial condyles of knee osteoarthritis patients**

Clara Sanjurjo-Rodriguez^1,2^, Thomas G. Baboolal^1,3^, Agata N. Burska^1,^, Frederique Ponchel^1,^, Jehan J. El-Jawhari^1,4^, Hemant Pandit^1,3,5^, Dennis McGonagle^1,3,5^, Elena Jones^1,^*.

**Supplementary materials and methods**

**Angiogenesis tube formation assay**

To compare the paracrine angiogenic potential of MSCs from medial and lateral condyles, Matrigel-based angiotube formation assay was performed as previously described^1^. Briefly, MSCs were first seeded into 6 well plates containing StemMACS media and upon reaching 80% confluence, transferred into DMEM media supplemented with 1% FBS. Culture supernatants were collected after 72 hours, centrifuged at 300 x g for 10 minutes to eliminate cell debris and frozen at -80ºC until use. For the angiogenesis tube assay, 96-well culture plates (Corning) were pre-coated with 50 µl Matrigel (Corning Inc.), on top of which 10^4^ human umbilical vein endothelial cells (HUVECs; Lonza, Switzerland) were seeded with the addition of MSC culture supernatants. Basal DMEM/1% FBS medium was used as negative control, and HUVEC medium (Endothelial Cell Growth Medium 2; PromoCell, Germany) was used as positive control. All conditions were assessed in duplicate. Plates were placed in the incubator at 37ºC and images were taken after three hours. Tube lengths was measured using the Angiogenic analyzer plug-in for ImageJ, as described before^2^.

**Tables**

**Supplementary Table 1:** Genes differentially expressed between multipotential stromal cells (MSC) and chondrocytes (CH).

| **Transcript** | **MSC/CH median fold-difference** | **p-value** |  | **Transcript** | **CH/MSC median fold-difference** | **p-value** |
| --- | --- | --- | --- | --- | --- | --- |
| SP7 | CH BD | NA |  | COMP | MSC BD | NA |
| HGF | CH LD | NA |  | CCL20 | MSC BD | NA |
| ARNTL | CH LD | NA |  | NOS2 | MSC BD | NA |
| IBSP | 1020.3 | <0.0001 |  | CCR7 | MSC BD | NA |
| IGF2 | 103.8 | <0.0001 |  | LCN2 | MSC LD | NA |
| CXCL12 | 60.3 | <0.0001 |  | IGF1 | MSC LD | NA |
| RUNX2 | 53.3 | <0.0001 |  | IL10 | MSC LD | NA |
| TNFSF11 | 29.7 | <0.0001 |  | MMP3 | 105.2 | <0.0001 |
| MMP9 | 19.6 | <0.0001 |  | FABP4 | 25.7 | <0.0001 |
| COL1A1 | 13.3 | <0.0001 |  | MMP1 | 21.4 | <0.0001 |
| MMP13 | 11.8 | 0.0012 |  | CCL5 | 17.1 | 0.0004 |
| STMN2 | 10.1 | 0.0017 |  | DIO2 | 14.2 | <0.0001 |
| TIMP3 | 8.0 | <0.0001 |  | CCR1 | 12.6 | 0.0016 |
| TGFB1 | 6.9 | <0.0001 |  | IGFBP3 | 4.4 | <0.0001 |
| LEPR | 6.4 | <0.0001 |  | ADAMTS5 | 3.4 | <0.0001 |
| SPHK1 | 6.3 | <0.0001 |  | CCL2 | 3.1 | <0.0001 |
| S1PR1 | 5.8 | <0.0001 |  | NGF | 2.3 | 0.0364 |
| NOTCH1 | 4.8 | <0.0001 |  | PTH1R | 2.1 | 0.0048 |
| MMP2 | 4.1 | <0.0001 |  |  |  |  |
| ADAMTS4 | 4.1 | <0.0001 |  |  |  |  |
| PPARd | 3.9 | <0.0001 |  |  |  |  |
| COL1A2 | 3.8 | <0.0001 |  |  |  |  |
| TGFBR1 | 3.7 | <0.0001 |  |  |  |  |
| SFRP1 | 3.6 | 0.0071 |  |  |  |  |
| WISP1 | 3.2 | <0.0001 |  |  |  |  |
| ANKH | 3.0 | <0.0001 |  |  |  |  |
| IGF1R | 2.7 | <0.0001 |  |  |  |  |
| SPARC | 2.5 | 0.0021 |  |  |  |  |
| POSTN | 2.4 | 0.0005 |  |  |  |  |
| GREM1 | 2.1 | 0.0450 |  |  |  |  |

BD, below detection (in all donors); LD, low detection (detected in <50% donors); NA, not applicable. Fold-difference is calculated based on the median values for each group, Mann-Whitney test used for groups’ comparisons.

**Supplementary Table 2:** Genes differentially expressed between CD271^+^CD45^-^ multipotential stromal cells (MSC) and CD271^-^CD45^+^ haematopoietic lineage cells (HLC).

| **Transcript** | **HLC/MSC median fold-difference** | **p-value** |  | **Transcript** | **MSC/HLC median fold-difference** | **p-value** |
| --- | --- | --- | --- | --- | --- | --- |
| CCR5 | BD in MSC | NA |  | SP7 | BD in HLC | NA |
| CCR2 | BD in MSC | NA |  | SFRP1 | BD in HLC | NA |
| CCR6 | BD in MSC | NA |  | STMN2 | BD in HLC | NA |
| DIRAS2 | BD in MSC | NA |  | LEPR | LD in HLC | NA |
| CCR1 | LD in MSC | NA |  | NGF | LD in HLC | NA |
| IL1B | 1346.9 | <0.0001 |  | WISP1 | LD in HLC | NA |
| MMP9 | 662.7 | <0.0001 |  | THBS4 | LD in HLC | NA |
| IL10 | 389.9 | <0.0001 |  | MMP13 | LD in HLC | NA |
| CCL5 | 212.6 | <0.0001 |  | ASPN | LD in HLC | NA |
| CXCR4 | 135.6 | <0.0001 |  | ROR2 | LD in HLC | NA |
| CCR7 | 49.6 | <0.0001 |  | PTH1R | 468.4 | <0.0001 |
| TNFa | 7.9 | 0.0043 |  | SFRP4 | 359.8 | <0.0001 |
| TGFB1 | 4.8 | <0.0001 |  | IGF2 | 326.8 | <0.0001 |
| CCL20 | 3.5 | 0.0015 |  | DIO2 | 194.9 | <0.0001 |
| TIMP1 | 2.2 | <0.0001 |  | SOX9 | 166.0 | <0.0001 |
|  |  |  |  | ADAMTS5 | 137.4 | <0.0001 |
|  |  |  |  | CTGF | 119.2 | <0.0001 |
|  |  |  |  | CYR61 | 101.7 | <0.0001 |
|  |  |  |  | VEGFC | 100.1 | <0.0001 |
|  |  |  |  | NGFR | 99.3 | <0.0001 |
|  |  |  |  | HGF | 95.3 | <0.0001 |
|  |  |  |  | CXCL12 | 94.4 | <0.0001 |
|  |  |  |  | DDR2 | 86.3 | <0.0001 |
|  |  |  |  | IBSP | 70.7 | <0.0001 |
|  |  |  |  | MMP3 | 66.2 | <0.0001 |
|  |  |  |  | IGFBP3 | 63.4 | <0.0001 |
|  |  |  |  | MMP2 | 50.3 | <0.0001 |
|  |  |  |  | ADAMTS4 | 40.4 | <0.0001 |
|  |  |  |  | COL1A2 | 39.1 | <0.0001 |
|  |  |  |  | POSTN | 37.1 | 0.0004 |
|  |  |  |  | TIMP3 | 36.8 | <0.0001 |
|  |  |  |  | TGFB2 | 35.2 | <0.0001 |
|  |  |  |  | TNFSF11 | 31.5 | <0.0001 |
|  |  |  |  | COL1A1 | 31.5 | <0.0001 |
|  |  |  |  | TGFB3 | 24.3 | <0.0001 |
|  |  |  |  | SERPINE1 | 13.9 | <0.0001 |
|  |  |  |  | IL6 | 13.7 | <0.0001 |
|  |  |  |  | SPARC | 12.7 | <0.0001 |
|  |  |  |  | CCL2 | 10.2 | <0.0001 |
|  |  |  |  | PPARg | 10.2 | <0.0001 |
|  |  |  |  | PTHLH | 8.2 | 0.0003 |
|  |  |  |  | TIMP2 | 4.7 | <0.0001 |
|  |  |  |  | SPP1 | 4.0 | 0.0039 |
|  |  |  |  | PTGS2 | 3.6 | <0.0001 |
|  |  |  |  | IGF1R | 3.6 | <0.0001 |
|  |  |  |  | RUNX2 | 3.3 | <0.0001 |
|  |  |  |  | ANKH | 3.2 | <0.0001 |
|  |  |  |  | MMP14 | 3.0 | <0.0001 |
|  |  |  |  | TGFBR1 | 2.9 | 0.0129 |
|  |  |  |  | ACAN | 2.8 | 0.0400 |
|  |  |  |  | MMP1 | 2.7 | 0.0129 |
|  |  |  |  | VEGFA | 2.5 | <0.0001 |
|  |  |  |  | S1PR1 | 2.2 | 0.0015 |
|  |  |  |  | FABP4 | 2.2 | 0.0301 |
|  |  |  |  | SPHK1 | 2.1 | 0.0107 |

BD, below detection (in all donors); LD, low detection (detected in <50% donors). Fold-difference is calculated based on the median values for each group, Mann-Whitney test used for groups’ comparisons.

**Supplementary table 3:** Taqman probes used to assess the gene expression.

| **Gene symbol** | **Gene name** | **Assay number** | **Bibliography** |
| --- | --- | --- | --- |
| ACAN | aggrecan | HS00153936-m1 | ^3^, ^4^, ^5^, ^6^, ^7^ |
| ADAMTS4 | a disintegrin and metalloproteinase with thrombospondin motifs 4 | HS00192708-m1 | ^3^, ^4^, ^6^, ^8^, ^9^ |
| ADAMTS5 | a disintegrin and metalloproteinase with thrombospondin motifs 5 | HS01095524-m1 | ^3^, ^4^, ^6^, ^8^, ^10^, ^9^, ^11^ |
| ANKH | human homolog of the murine progressive ankylosis gene | HS01064613-m1 | ^3^ |
| ARNTL | aryl hydrocarbon receptor nuclear translocator-like protein 1 | HS00154147-m1 | ^12^ |
| ASPN | asporin | HS01558901-m1 | ^13^, ^6^ |
| BCL2 | BCL2, apoptosis regulator | HS00608023-m1 | ^14^, ^15^ |
| BGLAP | Bone Gamma-Carboxyglutamate Protein | HS01587814-g1 | ^3^,^16^ |
| BMPR1B | Bone Morphogenetic Protein Receptor Type 1B | HS01010965-m1 | ^17^ |
| CCL19 | C-C Motif Chemokine Ligand 19 | HS00171149-m1 | ^8^ |
| CCL2 | C-C Motif Chemokine Ligand 2 | HS00234140-m1 | ^13^, ^6^, ^18^, ^19^ |
| CCL20 | C-C Motif Chemokine Ligand 20 | HS01011368-m1 | ^6^ |
| CCL5 | C-C Motif Chemokine Ligand 5 | HS00982282-m1 | ^8^, ^19^ |
| CCR1 | C-C Motif Chemokine Receptor 1 | HS00174298-m1 | ^20^ |
| CCR10 | C-C Motif Chemokine Receptor 10 | HS00706455-s1 | ^20^ |
| CCR2 | C-C Motif Chemokine Receptor 2 | HS00356601-m1 | ^6^, ^19^ |
| CCR3 | C-C Motif Chemokine Receptor 3 | HS99999027-s1 | ^20^ |
| CCR5 | C-C motif chemokine receptor 5 | HS00152917-m1 | ^19^ |
| CCR6 | C-C Motif Chemokine Receptor 6 | HS00171121-m1 | ^6^ |
| CCR7 | C-C Motif Chemokine Receptor 7 | HS04398702-m1 | ^20^ |
| COL10A1 | Collagen Type X Alpha 1 Chain | HS00166657-m1 | ^4^,^5^, ^6^, ^9^, ^17^ |
| COL1A1 | Collagen Type I Alpha 1 Chain | HS01076777-m1 | ^3^, ^7^ |
| COL1A2 | Collagen Type I Alpha 2 Chain | HS01028971-m1 | ^3^, ^6^, ^7^ |
| COL2A1 | Collagen Type II Alpha 1 Chain | HS00264051-m1 | ^3^, ^4^, ^5^, ^10^, ^21^ |
| COMP | Cartilage Oligomeric Matrix Protein | HS00164359-m1 | ^3^, ^21^, ^7^ |
| CTGF | Connective Tissue Growth Factor | HS00170014-m1 | ^6^ |
| CXCL12 | C-X-C Motif Chemokine Ligand 12 | HS00171022-m1 | ^22^, ^6^, ^7^ |
| CXCR1 | C-X-C Motif Chemokine Receptor 1 | HS01921207-m1 | ^6^ |
| CXCR4 | C-X-C Motif Chemokine Receptor 4 | HS00607978_S1 | ^6^ |
| CYR61 | Cysteine Rich Angiogenic Inducer 61 | HS00155479-m1 | ^6^ |
| DDR2 | iscoidin Domain Receptor Tyrosine Kinase 2 | HS01025956-m1 | ^4^ |
| DIO2 | Iodothyronine Deiodinase 2 | HS00255341-m1 | ^13^, ^8^ |
| DIRAS2 | DIRAS Family GTPase 2 | HS01107862-m1 | ^23^ |
| FABP4 | Fatty Acid Binding Protein 4 | HS00609791-m1 | ^6^ |
| GDF5 | Growth Differentiation Factor 5 | HS00167060-m1 | ^3^, ^6^, ^8^, ^18^, ^17^, ^7^ |
| GDF6 | Growth Differentiation Factor 6 | HS01377663-m1 | ^3^, ^22^ |
| GREM1 | Gremlin 1, DAN Family BMP Antagonist | HS00171951-m1 | ^18^ |
| HGF | Hepatocyte Growth Factor | HS00300159-m1 | ^3^ |
| **HPRT1** | Hypoxanthine Phosphoribosyltransferase 1 | HS99999909-m1 | House-keeping gene |
| IBSP | integrin binding sialoprotein | HS00173720-m1 | ^3^ |
| IGF1 | Insulin Like Growth Factor 1 | HS03986524-m1 | ^3^, ^22^, ^5^, ^10^, ^24^, ^25^ |
| IGF1R | Insulin Like Growth Factor 1 Receptor | HS00609566-m1 | ^24^ |
| IGF2 | Insulin Like Growth Factor 2 | HS00171254-m1 | ^3^, ^22^, ^5^, ^26^ |
| IGFBP3 | Insulin Like Growth Factor Binding Protein 3 | HS00426289-m1 | ^27^, ^7^ |
| IL10 | Interleukin 10 | HS00961622-m1 | ^28^ |
| IL1B | Interleukin 1 Beta | HS01555413-m1 | ^3^, ^4^, ^5^, ^8^, ^28^, ^9^, ^11^ |
| IL6 | Interleukin 6 | HS00174131-m1 | ^3^, ^4^, ^5^, ^5^, ^8^, ^28^, ^9^, ^11^ |
| LCN2 | Lipocalin 2 | HS01008571-m1 | ^25^ |
| LEPR | Leptin Receptor | HS00174492-m1 | ^9^ |
| MMP1 | Matrix Metallopeptidase 1 | HS00899658-m1 | ^3^, ^4^, ^5^, ^6^, ^8^, ^9^ |
| MMP13 | Matrix Metallopeptidase 13 | HS00942589-m1 | ^23^, ^3^, ^4^, ^5^, ^6^, ^8^, ^10^, ^9^, ^11^ |
| MMP14 | Matrix Metallopeptidase 14 | HS00237119-m1 | ^21^ |
| MMP2 | Matrix Metallopeptidase 2 | HS01548728-m1 | ^3^, ^4^, ^5^, ^6^, ^21^ |
| MMP3 | Matrix Metallopeptidase 3 | HS00968308-m1 | ^3^, ^4^, ^22^, ^5^, ^6^, ^8^, ^9^ |
| MMP9 | Matrix Metallopeptidase 9 | HS00957562-m1 | ^3^, ^4^, ^5^, ^25^, ^9^, ^11^ |
| NGF | Nerve Growth Factor | HS00171458-m1 | ^6^, ^18^ |
| NGFR | Nerve Growth Factor Receptor | HS00182120-m1 | ^6^, ^18^ |
| NOS2 | Nitric Oxide Synthase 2 | HS01075529-m1 | ^3^, ^8^, ^9^, ^11^ |
| NOTCH1 | Notch 1 | HS01062014_m1 | ^11^ |
| POSTN | Periostin | HS01566750-m1 | ^13,22^ |
| PPARd | Peroxisome Proliferator Activated Receptor Delta | HS00602622-m1 | ^29^ |
| PPARg | Peroxisome Proliferator Activated Receptor Gamma | HS01115513-M1 | ^22^, ^6^, ^7^ |
| PSIP1 | PC4 And SFRS1 Interacting Protein 1 | HS01045714-g1 | ^23^ |
| PTGS2 | Prostaglandin-Endoperoxide Synthase 2 | HS00153133-m1 | ^30^ |
| PTH1R | Parathyroid Hormone 1 Receptor | HS00896824-m1 | ^15^, ^10^ |
| PTHLH | Parathyroid Hormone Like Hormone | HS00174969-m1 | ^5^, ^15^, ^10^, ^17^ |
| ROR2 | Receptor Tyrosine Kinase Like Orphan Receptor 2 | HS00171695-m1 | ^31^ |
| RUNX2 | Runt Related Transcription Factor 2 | HS00234692-m1 | ^3^, ^4^, ^6^, ^10^, ^17^ |
| S1PR1 | Sphingosine-1-Phosphate Receptor 1 | HS00173499-m1 | ^3^ |
| SERPINE1 | Serpin Family E Member 1 | HS00167155-m1 | ^26^ |
| SFRP1 | Secreted Frizzled Related Protein 1 | HS00610060-m1 | ^32^ |
| SFRP4 | Secreted Frizzled Related Protein 4 | HS00180066-m1 | ^7^ |
| SOX9 | SRY-Box 9 | HS00165814-m1 | ^4^, ^6^, ^17^, ^16^ |
| SP7 | Sp7 Transcription Factor | HS00541729-m1 | ^16^ |
| SPARC | Secreted Protein Acidic And Cysteine Rich | HS00277762-m1 | ^6^, ^7^ |
| SPHK1 | Sphingosine Kinase 1 | HS00184211-m1 | ^3^ |
| SPP1 | Secreted Phosphoprotein 1 | HS00959010-m1 | ^3^, ^4^ |
| STMN2 | Stathmin 2 | HS00975800-m1 | ^23^, ^18^ |
| TGFB1 | transforming growth factor beta 1 | HS00998133-m1 | ^3^, ^4^, ^22^, ^5^, ^6^, ^10^, ^25^ |
| TGFB2 | transforming growth factor beta 2 | HS00234244-m1 | ^3^, ^4^, ^22^, ^5^ |
| TGFB3 | Transforming Growth Factor Beta 3 | HS01085997-m1 | ^3^, ^4^, ^5^, ^21^ |
| TGFBR1 | Transforming Growth Factor Beta Receptor 1 | HS00610319-m1 | ^6^, ^10^ |
| TGFBR2 | transforming growth factor beta receptor 2 | HS00559661-m1 | ^6^, ^10^ |
| TGFBR3 | Transforming Growth Factor Beta Receptor 3 | HS00234257-m1 | ^10^ |
| THBS4 | Thrombospondin 4 | HS00170261-m1 | ^23^ |
| TIMP1 | TIMP metallopeptidase inhibitor 1 | HS00171558-m1 | ^6^, ^21^ |
| TIMP2 | TIMP metallopeptidase inhibitor 2 | HS01091319-m1 | ^6^, ^26^ |
| TIMP3 | TIMP Metallopeptidase Inhibitor 3 | HS00927214-m1 | ^6^, ^21^ |
| TNFa | Tumor Necrosis Factor | HS99999043-m1 | ^3^, ^13,22^, ^5^, ^8^, ^28^, ^11^ |
| TNFRSF11B | TNF Receptor Superfamily Member 11b | HS00900360-m1 | ^13^, ^5^, ^6^, ^10^ |
| TNFSF11 | TNF Superfamily Member 11 | HS01092186-m1 | ^3^, ^4^, ^13,22^, ^5^, ^6^, ^10^, ^32^ |
| VEGFA | Vascular Endothelial Growth Factor A | HS00900058-m1 | ^3^, ^4^, ^5^, ^6^, ^10^, ^11^ |
| VEGFC | Vascular Endothelial Growth Factor C | HS01099206-m1 | ^3^, ^5^, ^7^ |
| WISP1 | WNT1 Inducible Signaling Pathway Protein 1 | HS04234730-m1 | ^3^, ^13^ |
| WNT10b | Wnt Family Member 10B | HS00559664-m1 | ^13^ |

**Figures**

**Supplementary Figure 1**

**
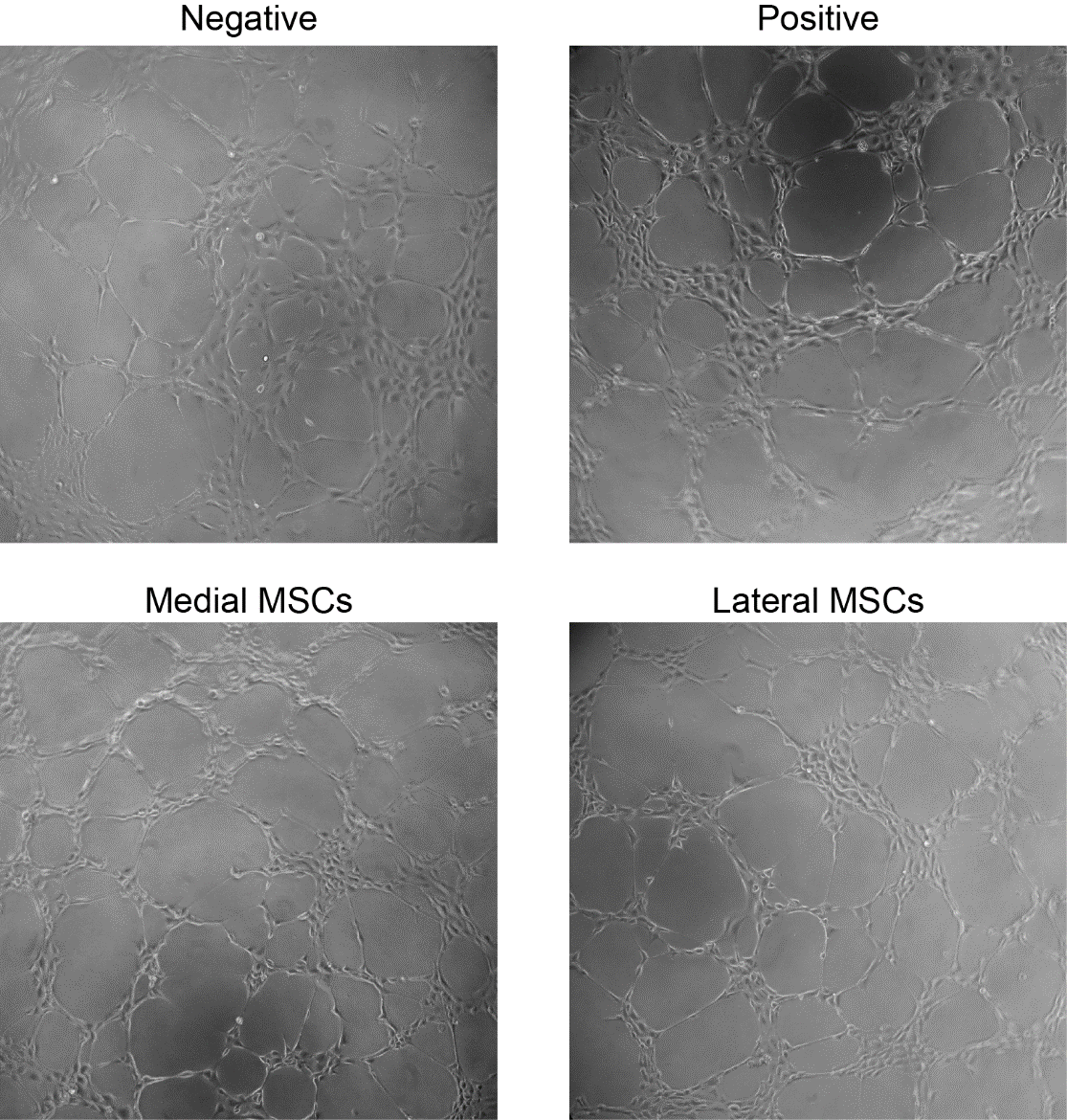
**

**Supplementary figure 1:** Representative images illustrating angiogenic tube formation assay in Matrigel. Negative: human umbilical vein endothelial cells (HUVECs) seeded in DMEM supplemented with 1% FCS; Positive: HUVECs seeded in HUVEC medium (Endothelial Cell Growth Medium 2), more tubes and junctions are evident; Medial MSCs: HUVECs seeded in conditioned-medium DMEM 1% FCS obtained from medial condyle MSCs; Lateral MSCs: HUVECs seeded in conditioned-medium DMEM 1% FCS obtained from lateral condyle MSCs. No notable differences in the number of tubes and junctions are evident between media conditioned by medial and lateral condyle MSCs.

**Supplementary Figure 2**

**
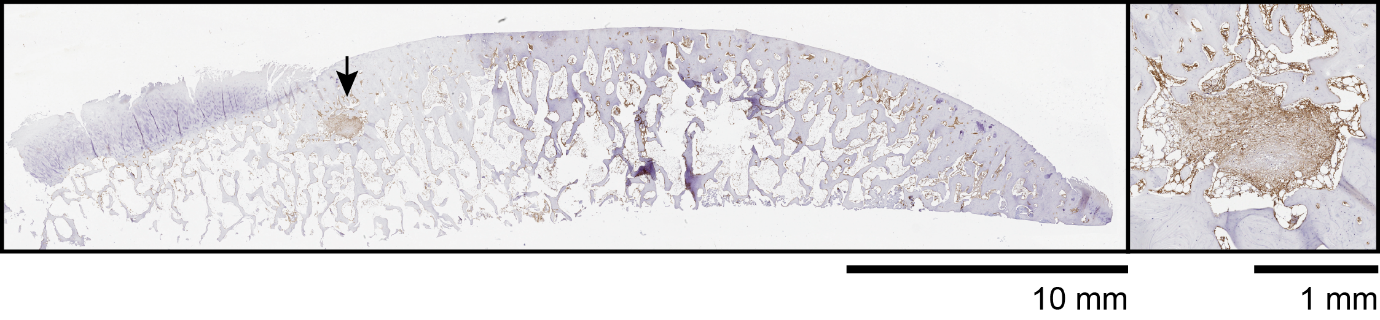
**

**Supplementary figure 2:** Immunohistochemistry showing CD271 positivity in stromal tissue of a cyst (arrow and magnified image) within the medial condyle of OA donor tissue.

**Supplementary Figure 3**

**
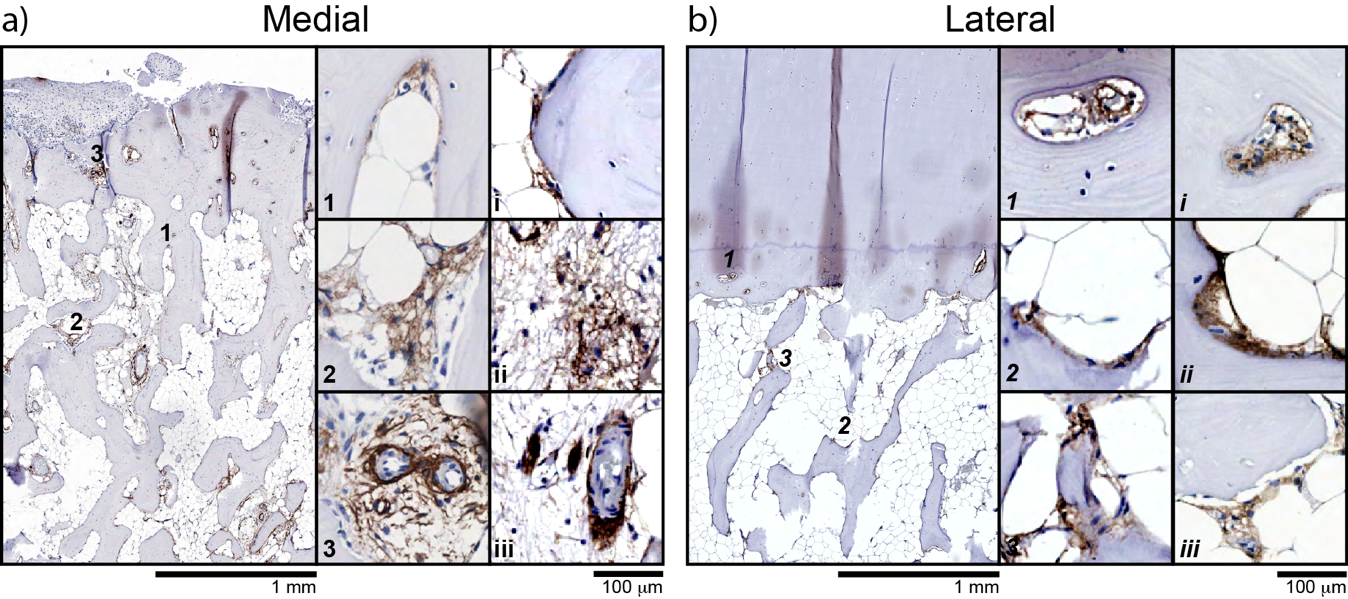
**

**Supplementary Figure 3:** Immunohistochemistry staining showing CD271 positivity within medial (a) and lateral (b) tibial plateau sections. a) Medial tibial plateau sections show region of cartilage denudation with CD271 positivity on bone lining cells (1 and i), positivity in stromal tissue (2 and ii) and around blood vessels (3 and iii). Magnified images show corresponding (1-3) areas and similar features from other donors shown in (i-iii). b) Lateral tibial plateau images showing areas of CD271 positivity in the presence of a full thick cartilage layer. Positivity was seen in areas of vascular channel invasion (*1* and *i*) and characteristic bone lining MSCs (*2-3* and *ii-iii*). Magnified images show corresponding *(1-3)* areas and similar features from other donors shown in *(i-iii)*.

**Supplementary references**

1. Bejar, M.T., Ferrer-Lorente, R., Peña, E. & Badimon, L. Inhibition of Notch rescues the angiogenic potential impaired by cardiovascular risk factors in epicardial adipose stem cells. *FASEB J* **30**, 2849-2859 (2016).

2. Khoo, C.P., Micklem, K. & Watt, S.M. A comparison of methods for quantifying angiogenesis in the Matrigel assay in vitro. *Tissue Eng Part C Methods* **17**, 895-906 (2011).

3. Sharma, A.R., Jagga, S., Lee, S.S. & Nam, J.S. Interplay between cartilage and subchondral bone contributing to pathogenesis of osteoarthritis. *Int J Mol Sci* **14**, 19805-19830 (2013).

4. Goldring, S.R. & Goldring, M.B. Changes in the osteochondral unit during osteoarthritis: structure, function and cartilage-bone crosstalk. *Nat Rev Rheumatol* **12**, 632-644 (2016).

5. Karsdal, M.A.*, et al.* The coupling of bone and cartilage turnover in osteoarthritis: opportunities for bone antiresorptives and anabolics as potential treatments? *Ann Rheum Dis* **73**, 336-348 (2014).

6. Campbell, T.M.*, et al.* Mesenchymal Stem Cell Alterations in Bone Marrow Lesions in Patients With Hip Osteoarthritis. *Arthritis Rheumatol* **68**, 1648-1659 (2016).

7. Baboolal, T.G.*, et al.* Intrinsic multipotential mesenchymal stromal cell activity in gelatinous Heberden's nodes in osteoarthritis at clinical presentation. *Arthritis Res Ther* **16**, R119 (2014).

8. Martel-Pelletier, J.*, et al.* Osteoarthritis. *Nat Rev Dis Primers* **2**, 16072 (2016).

9. Scotece, M. & Mobasheri, A. Leptin in osteoarthritis: Focus on articular cartilage and chondrocytes. *Life Sci* **140**, 75-78 (2015).

10. Zhen, G. & Cao, X. Targeting TGFbeta signaling in subchondral bone and articular cartilage homeostasis. *Trends Pharmacol Sci* **35**, 227-236 (2014).

11. Saito, T. & Tanaka, S. Molecular mechanisms underlying osteoarthritis development: Notch and NF-kappaB. *Arthritis Res Ther* **19**, 94 (2017).

12. Dudek, M.*, et al.* The chondrocyte clock gene Bmal1 controls cartilage homeostasis and integrity. *J Clin Invest* **126**, 365-376 (2016).

13. Chou, C.H.*, et al.* Direct assessment of articular cartilage and underlying subchondral bone reveals a progressive gene expression change in human osteoarthritic knees. *Osteoarthritis Cartilage* **21**, 450-461 (2013).

14. Zaman, F.*, et al.* Dexamethasone differentially regulates Bcl-2 family proteins in human proliferative chondrocytes: role of pro-apoptotic Bid. *Toxicol Lett* **224**, 196-200 (2014).

15. Bovee, J.V., Cleton-Jansen, A.M., Taminiau, A.H. & Hogendoorn, P.C. Emerging pathways in the development of chondrosarcoma of bone and implications for targeted treatment. *Lancet Oncol* **6**, 599-607 (2005).

16. Chen, Y.*, et al.* Bone turnover and articular cartilage differences localized to subchondral cysts in knees with advanced osteoarthritis. *Osteoarthritis Cartilage* **23**, 2174-2183 (2015).

17. Wu, L.*, et al.* Human developmental chondrogenesis as a basis for engineering chondrocytes from pluripotent stem cells. *Stem Cell Reports* **1**, 575-589 (2013).

18. McGonagle, D., Baboolal, T.G. & Jones, E. Native joint-resident mesenchymal stem cells for cartilage repair in osteoarthritis. *Nature Reviews Rheumatology* **13**, 719-+ (2017).

19. Raghu, H.*, et al.* CCL2/CCR2, but not CCL5/CCR5, mediates monocyte recruitment, inflammation and cartilage destruction in osteoarthritis. *Ann Rheum Dis* **76**, 914-922 (2017).

20. Bernardini, G., Benigni, G., Scrivo, R., Valesini, G. & Santoni, A. The Multifunctional Role of the Chemokine System in Arthritogenic Processes. *Curr Rheumatol Rep* **19**, 11 (2017).

21. Liu, H.*, et al.* Enhanced tissue regeneration potential of juvenile articular cartilage. *Am J Sports Med* **41**, 2658-2667 (2013).

22. Chou, C.H.*, et al.* Insights into osteoarthritis progression revealed by analyses of both knee tibiofemoral compartments. *Osteoarthritis Cartilage* **23**, 571-580 (2015).

23. Kuttapitiya, A.*, et al.* Microarray analysis of bone marrow lesions in osteoarthritis demonstrates upregulation of genes implicated in osteochondral turnover, neurogenesis and inflammation. *Ann Rheum Dis* **76**, 1764-1773 (2017).

24. Heilig, J., Paulsson, M. & Zaucke, F. Insulin-like growth factor 1 receptor (IGF1R) signaling regulates osterix expression and cartilage matrix mineralization during endochondral ossification. *Bone* **83**, 48-57 (2016).

25. Villalvilla, A.*, et al.* The adipokine lipocalin-2 in the context of the osteoarthritic osteochondral junction. *Sci Rep* **6**, 29243 (2016).

26. Sanchez, C.*, et al.* Comparison of secretome from osteoblasts derived from sclerotic versus non-sclerotic subchondral bone in OA: A pilot study. *PLoS One* **13**, e0194591 (2018).

27. Evans, D.S.*, et al.* Genome-wide association and functional studies identify a role for IGFBP3 in hip osteoarthritis. *Ann Rheum Dis* **74**, 1861-1867 (2015).

28. Wojdasiewicz, P., Poniatowski, L.A. & Szukiewicz, D. The role of inflammatory and anti-inflammatory cytokines in the pathogenesis of osteoarthritis. *Mediators Inflamm* **2014**, 561459 (2014).

29. Ratneswaran, A.*, et al.* Peroxisome proliferator-activated receptor delta promotes the progression of posttraumatic osteoarthritis in a mouse model. *Arthritis Rheumatol* **67**, 454-464 (2015).

30. Pountos, I.*, et al.* NSAIDS inhibit in vitro MSC chondrogenesis but not osteogenesis: implications for mechanism of bone formation inhibition in man. *J Cell Mol Med* **15**, 525-534 (2011).

31. Sammar, M., Sieber, C. & Knaus, P. Biochemical and functional characterization of the Ror2/BRIb receptor complex. *Biochem Biophys Res Commun* **381**, 1-6 (2009).

32. Thysen, S., Luyten, F.P. & Lories, R.J. Loss of Frzb and Sfrp1 differentially affects joint homeostasis in instability-induced osteoarthritis. *Osteoarthritis Cartilage* **23**, 275-279 (2015).
